# A Review on the Fabrication of Polymer-Based Thermoelectric Materials and Fabrication Methods

**DOI:** 10.1155/2013/713640

**Published:** 2013-11-12

**Authors:** Muhammad Akmal Kamarudin, Shahrir Razey Sahamir, Robi Shankar Datta, Bui Duc Long, Mohd Faizul Mohd Sabri, Suhana Mohd Said

**Affiliations:** ^1^Department of Electrical Engineering, Faculty of Engineering, University of Malaya, 50603 Kuala Lumpur, Malaysia; ^2^Department of Mechanical Engineering, Faculty of Engineering, University of Malaya, 50603 Kuala Lumpur, Malaysia

## Abstract

Thermoelectricity, by converting heat energy directly into useable electricity, offers a promising technology to convert heat from solar energy and to recover waste heat from industrial sectors and automobile exhausts. In recent years, most of the efforts have been done on improving the thermoelectric efficiency using different approaches, that is, nanostructuring, doping, molecular rattling, and nanocomposite formation. The applications of thermoelectric polymers at low temperatures, especially conducting polymers, have shown various advantages such as easy and low cost of fabrication, light weight, and flexibility. In this review, we will focus on exploring new types of polymers and the effects of different structures, concentrations, and molecular weight on thermoelectric properties. Various strategies to improve the performance of thermoelectric materials will be discussed. In addition, a discussion on the fabrication of thermoelectric devices, especially suited to polymers, will also be given. Finally, we provide the challenge and the future of thermoelectric polymers, especially thermoelectric hybrid model.

## 1. Introduction

Global energy uncertainty and the limited resources coupled with increased energy demands provide the impetus for improving the efficiency of energy conversion technologies [1]. Therefore, the requirements of materials and technologies had been focused on those that contribute to energy conservation, safety, and environmental protection which lower the emission of CO_2_ [2]. Thermoelectricity, by converting heat energy directly into useable electricity, offers a promising technology to convert heat from the sun and to recover waste heat from industrial sectors and automobile exhausts [1–3]. The thermoelectric effect was first discovered by Thomas Seebeck in 1821 [1] when he discovered that twisting two wires together and twisting one end induced a voltage. The converse effect, that is, application of voltage to induce a temperature gradient across the thermoelectric material, was discovered by Jean Peltier [1] in 1834, and is thus called the Peltier effect. 

The performance of the thermoelectric material is evaluated by the dimensionless figure of merit (ZT), ZT = *σS* ^2^ *T*/*K*, where *σ* is the electrical conductivity, *K* is the thermal conductivity, *T* is the absolute temperature, and *S* is the Seebeck coefficient (*S* = Δ*V*/Δ*T*, that is, the ratio of the induced voltage over the temperature gradient across the thermoelectric device) [2]. Thus, a high performance thermoelectric material requires high electrical conductivity, Seebeck coefficient, and low thermal conductivity. It is really challenging to find a material which has a high Seebeck coefficient (*S*) in combination with high electrical conductivity (*σ*) and low thermal conductivity (*k*). In most materials, the electrical conductivity is directly proportional to the thermal conductivity. An ideal TE material would possess a high Seebeck coefficient as in the crystalline semiconductor, high electrical conductivity as in the crystalline metal, and low absolute temperature as in glass [4]. For practical applications, such as thermal generators (TEGs), a ZT of more than 3 is required, whilst the best efforts currently only produce a ZT of 3 [5]. 

Early thermoelectrical devices developed in the early 1960s earned some popularity given the solid state nature of the devices, that is, no moving parts compared to generators and motors. These devices were mainly based on Bi_2_Te_3_ [6, 7]. However, these devices have low efficiency (ZT < 1, and a system efficiency of <10%) and are therefore not cost-effective in most applications. In the mid-1990s, a research on thermoelectric started to gain interest again after theoretical predictions suggested that the thermoelectric efficiency could be enhanced through nanostructuring [8]. The introduction of nanostructures in thermoelectric materials served either to increase the electrical conductivity (through quantum dots), or to decrease the thermal conductivity (through nanowires and amorphous structures) [9–11]. Currently, the TE communication has paid the most attention on skutterudites [12], half-Heusler alloys [13], clathrates [14], and pentafluoriade [15]. The common characteristic of these materials is their complex structure, which serves to reduce the thermal conductivity and hence increase the ZT. These materials are usually targeted for high temperature operation, such as electricity generation from waste heat of industrial sources, such as steel furnaces and aluminum melting. This is due to their optimal ZT in a temperature range of 600 K or higher. One of the semiconductor compounds from the chalcogenide family, Bi_2_Te_3_, has already been commercially used in Peltier cooler. In terms of efficiency, Bi_2_Te_3_ alloys are the best TE materials known [16], which are able to optimally operate close to room temperature. However, the use of these materials is limited due to their toxic nature and also Te, which is a rare earth metal, makes the production cost uneconomical [17].

Numerous research works have been carried out to address these issues by replacing the metal- and alloy-based TE materials with organic and polymer materials [18, 19]. However, these materials have poor heat resistance which is unsuitable to be operated at high temperature. These polymer materials also have inferior thermoelectric properties than those of inorganic materials. To realistically apply these polymer materials for thermoelectric applications, applications which operate in the low temperature range (<100°C) need to be targeted. There are numerous polymer-based candidates for thermoelectric applications, which are easy to synthesize and fabricate low cost and low thermal conductivity.

In this paper, a review on the use of polymers in thermoelectric materials and devices will be given. The effect of different polymer structures, molecular concentration, and weight on thermoelectric properties will also be highlighted. Next, useful fabrication methods for solution-process-based fabrication, such as spin coating, inkjet printing, and electrospinning, shall be provided.

## 2. Polymer-Based Thermoelectric Materials

### 2.1. Advantages of Polymers in Thermoelectrics

Polymers as TE materials have attracted a lot of attention recently due to its easy fabrication processes and low material cost [20, 21]. Their physical and chemical properties can be tuned to the desired properties through simple molecular modifications, which allows for a large range of flexibility in polymer properties [22, 23]. In addition, carbon, which is the main element in polymers, is abundant in nature and thus the use of polymers in electronic devices is more economical and desirable. Polymers have a low thermal conductivity which proves to be desirable for TE applications. Examples of polymers that have been researched for TE applications are polyacetylene [24, 25], polypyrroles [26, 27], polyanilines [26, 28], polythiophenes [29, 30], and poly(2,7-carbazole)s [31, 32]. 

### 2.2. Factors Affecting the Thermoelectric Properties

#### 2.2.1. Various Polymer structures

**Figure 1:**
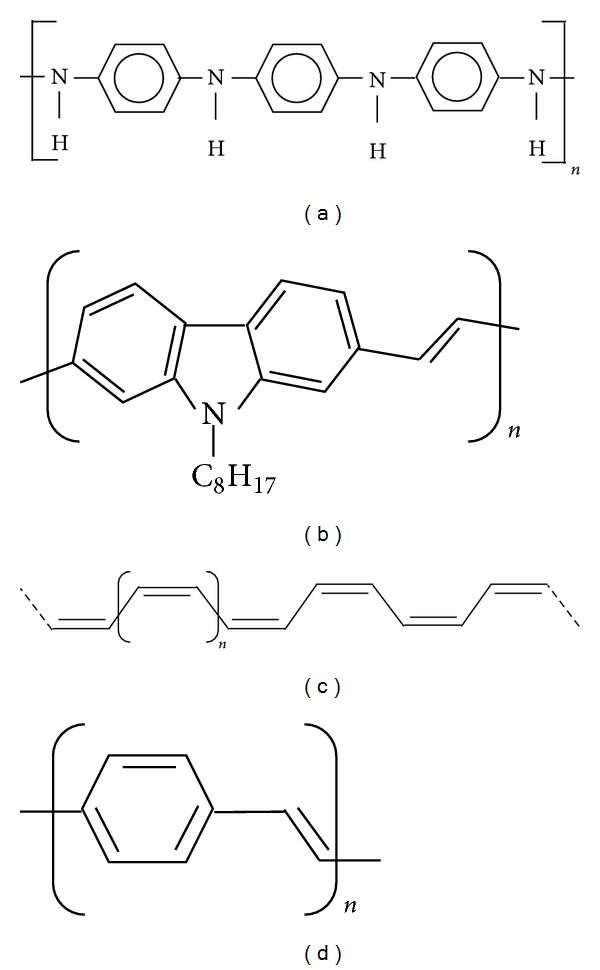
Various polymer structures of (a) polyaniline, (b) poly(2,7-carbazolenevinylene) [47], (c) polyacetylene and (d) poly(*p*-phenylene vinylene).

Different types of polymers have been used in thermoelectric devices, such as polyaniline (PANI) [41, 42], poly(*p*-phenylene vinylene) (PPV) [43, 44], polyacetylene (PA) [33, 34], poly(2,7-carbazolenevinylene) [32, 45], and poly(2,5-dimethoxy phenylenevinylene) (PMeOPV) [40]. These polymers are chosen due to their conductive nature. Different types of polymers (Figure 1 and Table 1) show different electrical conductivities, thermal conductivities, and figure of merits and exhibit various TE performances. In general, linear backbone polymers such as polyacetylene, polypyrrole, and polyaniline form the main class of conductive polymers. Poly(3-alkylthiophenes) form the basis for organic solar cells and transistors. The molecular basis of conductive properties lies in the conjugation of their bonds. Conductive polymers have continuous backbones of sp^2^ hybridised electrons, compared to the sp^3^ hybridised covalent bonds of nonconducting polymers. The valence electron of each sp^2^ hybridised carbon center combines a molecule wide delocalized orbital. These intrinsic organic semiconductors are typically doped oxidatively to form p-type organic semiconductors through dopants. Analogous to doping of inorganic intrinsic semiconductors, a small amount of doping (of around 0.1 wt%) causes the conductivity of the polymer to surge by 7–9 orders of magnitude. For example, undoped conjugated polymers such as polyacetylene may increase from 10^−10^ to 10^−8^ S/cm to around 0.1 S/cm upon doping. The frontrunner in the conductivity performance of conducting polymers is poly(3,4-ethylenedioxythiophene) (PEDOT), which, when doped with polystyrene sulfonate (PSS) or tosylate (Tos), may achieve conductivities of up to 3000 S/cm [46]. 

#### 2.2.2. Polymer Concentration

**Figure 2:**
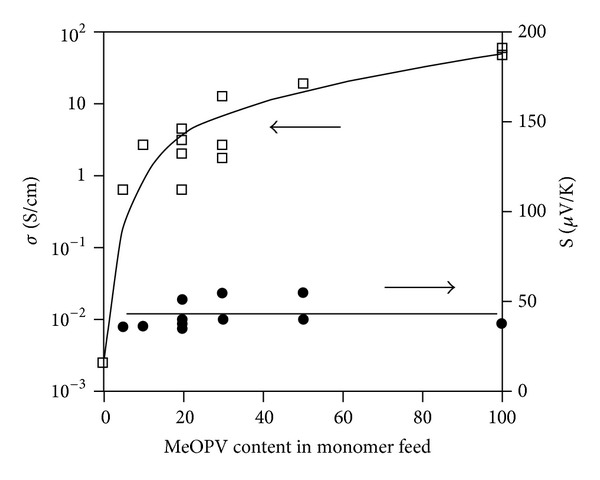
Conductivity dependence of monomer concentration [40].

Hiroshige et al. [40] showed that the electrical conductivity of poly (2,5-dimethoxy phenylenevinylene) (PMeOPV) increases with increasing monomer content of (methoxy phenylene vinylene) MeOPV whilst the Seebeck coefficient remains the same regardless of the monomer concentration. In their research, a relatively high Seebeck coefficient PMeOPV was observed at 39.1 *μ*V/K. To date, the thermal conductivity data and ZT of this material are not available. As the (methoxy phenylene vinylene) MeOPV content in the monomer feed is increased from 0 to 100 mol%, the conductivity increases from about 10^−3^ to 10^1^ S/cm.  Figure 2 shows the relationship of conductivity and monomer content. In the case of PEDOT : PSS blend [48], the trend is the same as the one described earlier. At high PSS content, PSS is the dominating factor and it is responsible for limiting the carrier transport within itself. But as the PSS content reduces, the distance between PEDOT : PSS cores effectively reduces as well and this affects the charge carrier mobility instead of affecting the charge carrier density. This is confirmed by the constant Seebeck coefficient even after reducing the PSS content. Similar observation has also been observed elsewhere [49]. 

#### 2.2.3. Polymer Molecular Weight and Chain Length

**Figure 3:**
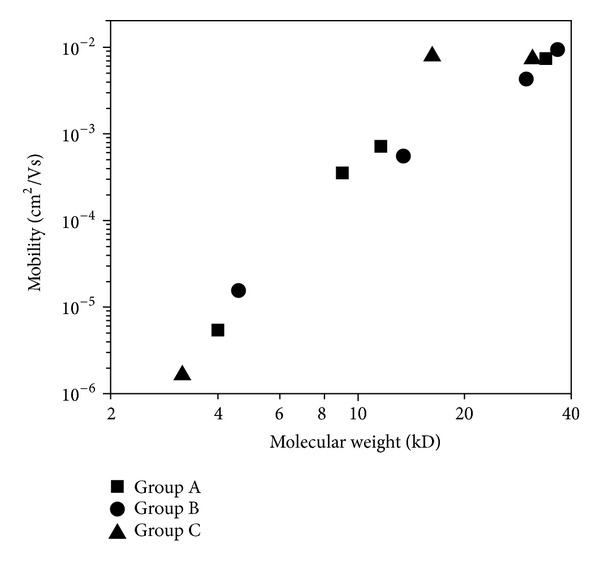
Field-effect mobility versus the number average molecular weight [50].

It is found that the molecular weights of the polymers have a substantial effect on the electron mobility and consequently affect the electrical conductivity. Kline et al. [50] found that an increase in chain length/molecular weight leads to the increase in electron mobility due to the fundamental mechanism of electrical conductivity in polymers, through electron hopping along the polymer backbone. A high molecular weight polymer will promote the charge carriers to move longer distances before hopping to another chain, and because longer chains give carriers more opportunities for hopping to neighboring chains. The decrease in the hopping could result in an increased mobility.  Figure 3 shows the polymer samples and their molecular weights which can be correlated with the electron mobility. Also, the thermal conductivity of polymer increases with the increase of polymer chain length as has been shown by Zhao et al. [51, 52]. 

#### 2.2.4. Temperature

**Figure 4:**
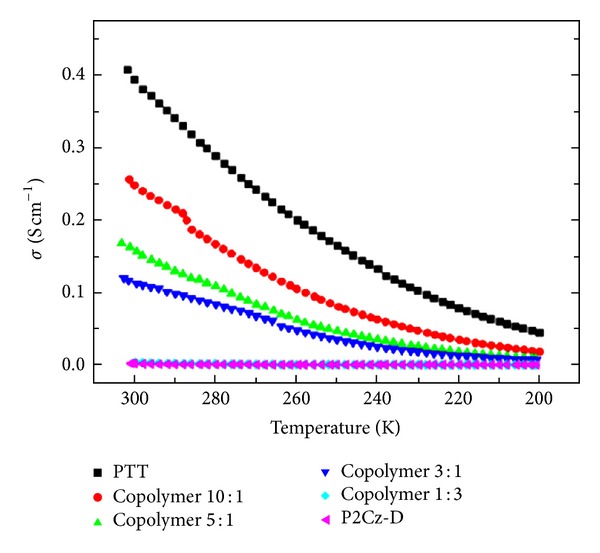
Temperature dependence of electrical conductivities for polythieno[3,2-b]thiophene (PTT), poly(1,12-bis(carbazolyl)dodecane) (P2Cz-D), and copolymers synthesized with monomer feed ratios of TT/2Cz-D = 10 : 1, 5 : 1, 3 : 1, and 1 : 3 in boron trifluoride diethyl etherate (BFEE) + dichloromethane (DCM) (30% vol) [19].

As a semiconducting polymers, the electrical conductivity increases with increasing temperature [19, 53, 54]. This is because when the temperature increases, the electrons that are charge carriers in the conductor will gain energy and jump up into higher energy levels. However, these energy levels are all still in the valence band. So the number of charge carriers will not change for a conductor with an increase in the temperature. On the other hand, the charge on each carrier and the electric field strength are not dependent on the temperature.  Figure 4 shows the temperature dependence of electrical conductivities of different types of polymers.

#### 2.2.5. Humidity

**Figure 5:**
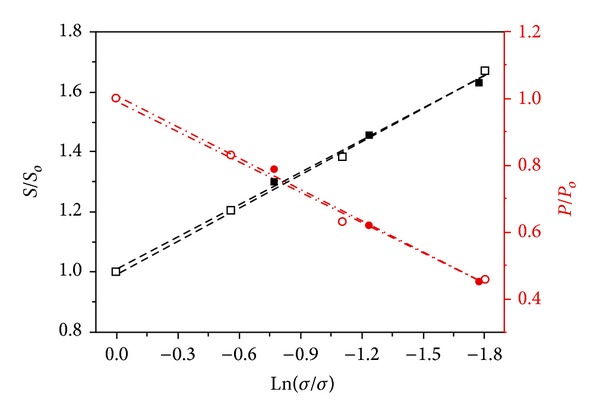
Thermoelectric properties of poly(3-hexylthiophene) P3HT-triflimide anion (TFSI) samples were filled and open symbols are samples that were kept inside and outside of desiccator, respectively [55].

Thermoelectric properties of conductive polymers tend to change with humidity. When humidity is high, the polymer forms carriers from the chemical dopants to result in high electrical conductivity. However, this also causes a corresponding decrease in the Seebeck coefficient. Thus, the improvement of the overall ZT is not significant. This demonstrates the difficulty in optimization of the ZT; that is, an increase in one parameter may actually cause a corresponding decrease in another parameter.  Figure 5 shows the effect of humidity (60%) on the conductivity and Seebeck coefficient [55]. 

#### 2.2.6. Alignment of Polymer Chains

**Figure 6:**
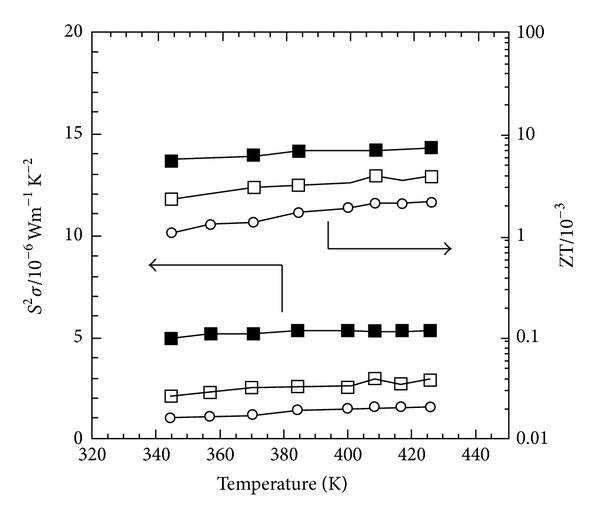
Thermoelectric power factor and ZT of CSA-doped stretched polyaniline films at various temperatures. Unstretched film (○), stretched film perpendicular (□), and parallel (■) to the stretching direction [59].

Alignment of the polymer chains is also known to increase the electrical conductivity of conductive polymers and thus increasing the ZT value [56, 57]. Toshima [58] found that the ZT value increased with increasing drawing ratio. Formation of a cast polymer is achieved through extrusion of a molten polymer through a flat die, onto a chill roll. Drawing ratio is defined as the velocity of the chill roll to the velocity of the polymer exiting the die onto an X-ray diffraction patterns and UV-Vis near IR spectra of a (±)-10-camphorsulfonic acid (CSA)-doped stretched polyaniline films suggested that the increase in ZT value can be attributed to the extended coil-like conformation that induced the alignment of polyaniline chains which increases the carrier mobility. It is also found that the stretching can increase the electrical conductivity parallel to the stretched direction twice that of the perpendicular [59]. Similarly, the Seebeck coefficient of the parallel stretched sample is also higher than that of the perpendicularly stretched sample, as shown in Figure 6. 

## 3. Strategies for Improvement of the Thermoelectric Performance

### 3.1. Doping

**Figure 7:**
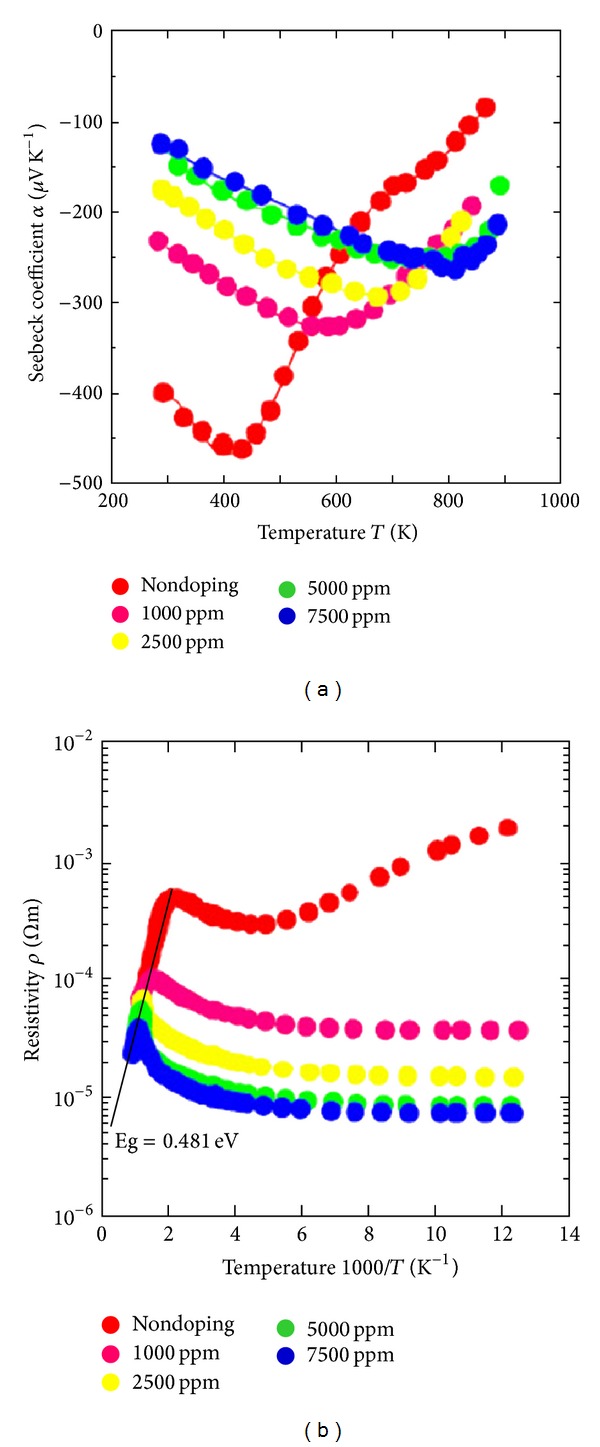
Seebeck coefficient and resistivity as a function of the doping level [65].

The electric conductivity can be improved by doping the polymers with a sufficient amount of suitable dopants. However, an increase in dopants leads to the decrease in the Seebeck coefficient. As the number of charge carriers increases, the Fermi energy is forced deep inside the conduction band [60]. Some examples of doping agents are iodine (I_2_) [61], Fe(III) Chloride (FeCl_3_) [31], molybdenum(V) chloride (MoCl_5_) [25], and so forth. It is found that an increase in the conductivity decreases the Seebeck coefficient [31, 62, 63].  Figure 7 shows the effect of doping on the Seebeck coefficient and resistivity.

In 2011, Bubnova et al. [54] optimized a PEDOT : Tos mixture to achieve a maximum ZT of 0.25 at room temperature. PEDOT : PSS is one of the most investigated organic semiconductor, having been extensively investigated for organic photovoltaic (OPV) and organic light emitting diode (OLED) applications, given its stability in air and high electrical conductivity. However, the performance of the PEDOT : PSS is somewhat hindered by the unionised portion of the dopants—typically the PSS dopant has a small ionization fraction, and the unionised portion reduces the carrier mobility and hence the overall thermoelectric power. Kim et al. [64] recently have optimized the ZT of the PEDOT : PSS mixture by removing the portion of unionized PSS dopant using a choice of solvents, thus achieving a record value of ZT = 0.42 for an organic thermoelectric polymer. 

### 3.2. Addition of Carbon Nanotubes (CNTs)

**Figure 8:**
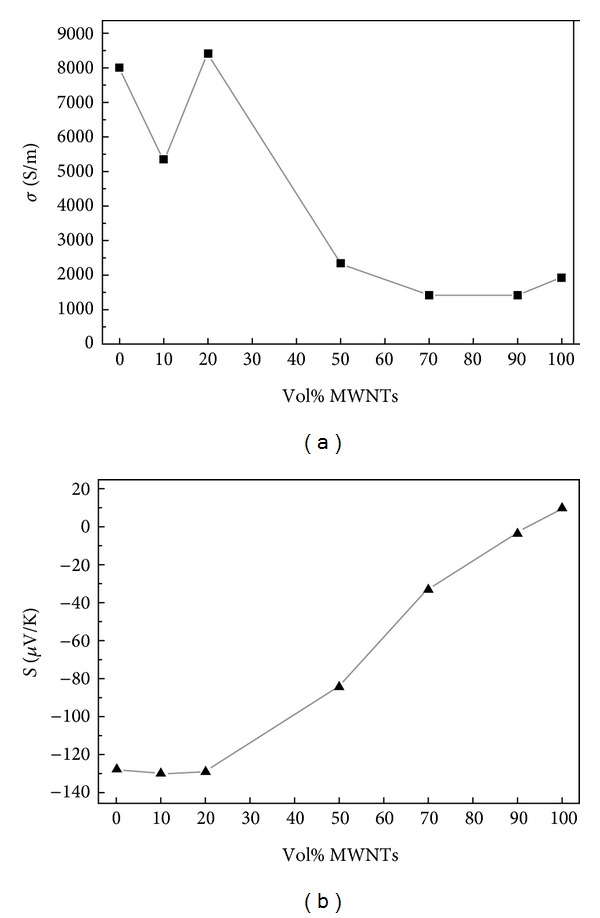
Effect of CNT doping on the electrical conductivity and Seebeck coefficient [67].

CNTs are known to have a stable one-dimensional nanostructure and excellent electrical and mechanical properties. When it is incorporated into a polymer matrix, the thermoelectric properties of the materials are affected. Kim et al. [66] found that PEDOT : PSS particles are spread on the surface of CNTs bridging tube-tube junction and allowing electrons to travel through the composite and thus increasing the electrical conductivity. On the other hand, heat transport in this system is suppressed due to differences in vibrational spectra between CNT and PEDOT : PSS. The highest ZT achieved was 0.02 when 35 wt% CNT was added to the polymer matrix. The addition of CNT as a dopant beyond a certain threshold also resulted in the decrease of both electrical and the Seebeck coefficient [67, 68]. This is due to the fact that CNTs act as impurities within the material and thus preventing the connection of adjacent particles which in turn greatly reduces the mean free path of the charge carriers. Also, an increase in CNT concentration will lead to the increase in the thermal conductivity [69].  Figure 8 shows the effect of CNT doping on the electrical conductivity and Seebeck coefficient.

### 3.3. Polymer Composite

Unlike CNTs, the addition of inorganic materials such as Te nanorods [70], Bi_2_Te_3_ [71], and Ca_3_Co_4_O_9_ [72] powders shows different effects. These materials basically have high Seebeck coefficient values. A research by See et al. [70] showed that by adding Te nanorods into a polymer matrix, the electrical conductivity increased, whereas the thermal conductivity decreased. Due to the Seebeck coefficient being positive and significantly higher than pristine polymer, it is thought that the holes were solely responsible for charge transport and that the transport did not occur exclusively through the polymer. The highest ZT achieved was ~0.1 at room temperature. 

### 3.4. Addition of Semiconducting Stabilizer

CNT is hydrophobic and tends to entangle in water which hinders complete dispersion and/or exfoliation in water [73, 74]. Stabilizing agents have been added to water-based composites containing CNT to make the composites more stable. Various types of stabilizers have been used to disperse CNT in water such as surfactants [75, 76], polymers [77, 78], and inorganic particles [79, 80]. The use of conductive polymer stabilizers especially can greatly improve the electrical conductivity [66] and hence increase thermopower.

**Figure 9:**
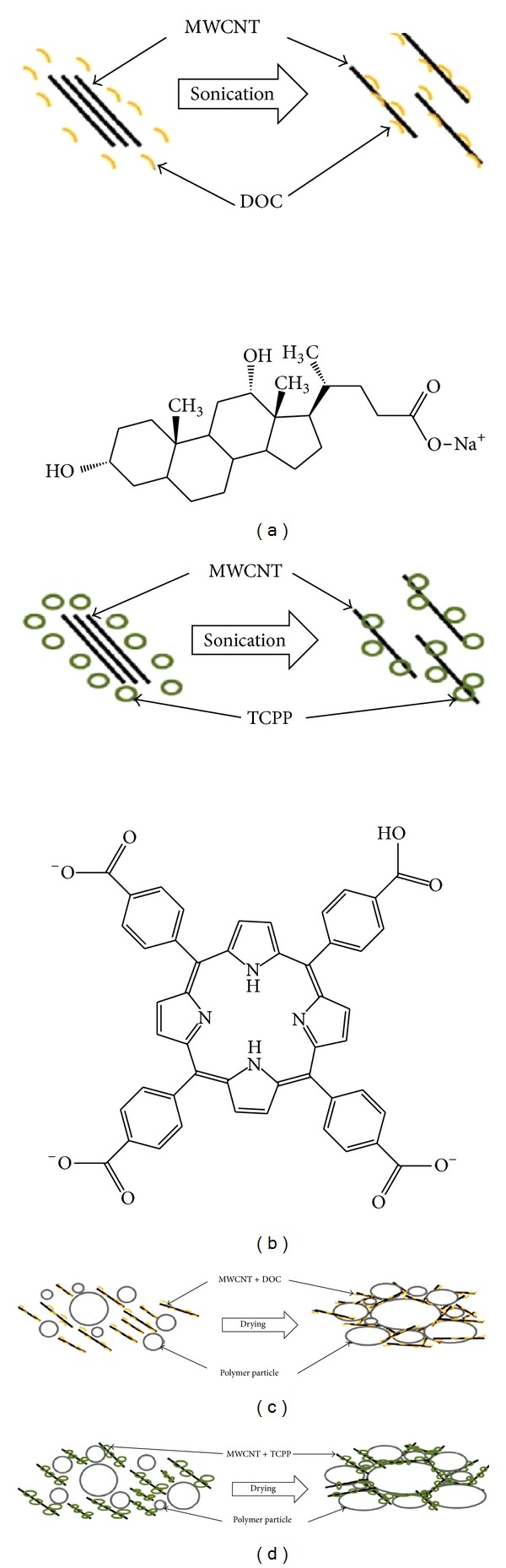
(a) and (b) show schematic diagram of carbon nanotubes dispersed in two different stabilizing agents, and (c) and (d) show the formation of network after water is dried out [81] where MWCNT: multi-walled carbon nanotube, TCPP: mesotetra(4-carboxyphenyl) porphine and DOC: sodium deoxycholate.

**Figure 10:**
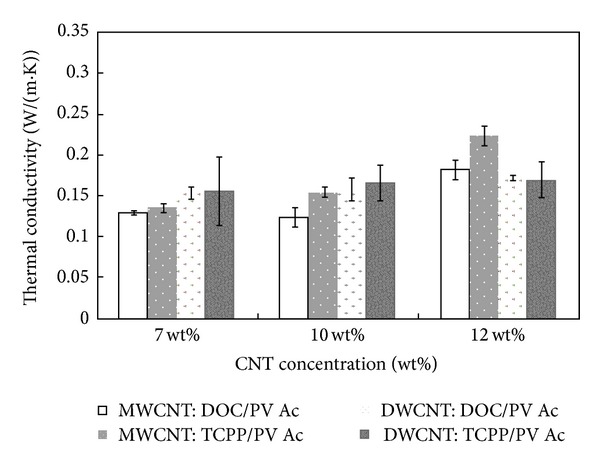
Thermal conductivity of sodium deoxycholate (DOC) and meso-tetra(4-carboxyphenyl)porphine (TCPP) stabilized systems containing different percentages of carbon nanotubes [81].

According to Moriarty et al. [81], the addition of stabilizers such as sodium deoxycholate (DOC) or meso-tetra(4-carboxyphenyl) porphine (TCPP) to CNT-polymer suspension suppresses the thermal conductivity by blocking tube-to-tube junctions which hinders phonon transport. The stabilizer also acts as phonon scattering centers since it is embedded in the composite alongside the CNT. The diameter and length of the tube, morphology of the CNTs, gaps between adjacent tubes, and defects introduced by the CNTs also contributed to the low thermal conductivity. Figures 9 and 10 show the effect of the addition of stabilizing agents on the interaction and thermal conductivity of the polymers.

### 3.5. Nanostructured Thermoelectric Materials

The use of nanofibers in thermoelectric devices can be potentially useful in achievement of a higher thermoelectric ZT. The reduced dimensionality of the nanofibers will mitigate thermal conductivity without any reduction in the electrical conductivity. This method is better than the use of bulk materials in terms of ZT and cost [82].

There is an extensive body of theoretical work concerned with efforts to improve ZT, through increase of electrical conductivity or decrease in thermal conductivity. Electrical conductivity may be improved through the use of quantum confinement effects, such as quantum dots, to enhance high electron density of states near the Fermi energy (*E*_*F*_). Conversely, thermal conductivity may be decreased through phonon blocking/scattering effects through the use of superlattices and nanowires. The ideal superlattice structure triggers phonon scattering and thereby reduces the thermal conductivity, whilst encouraging the motion of charge carriers through the superlattice [83]. 

The use of nanostructures also allows selective blocking of phonons whilst allowing transportation of charge carriers. This allows decoupling of thermal and electrical conductivity, which usually increase in tandem in normal conducting materials. This is attributed to the fact that if one increases the concentration of charge carriers in a thermoelectric medium, then this presents a higher possibility of collision of charge carriers with the crystal lattice, and hence thermal conductivity correspondingly increases. On the other hand, if the effective wavelengths of phonon and electrons are recognized and their effects separated, then the effects of thermal and electrical conductivity may be decoupled. The motion of phonons that carries most of the heat has mean free paths of hundreds of nanometers, whereas electrons have 10 nm or less. Hence, it is feasible to confine the movement of phonons without obstructing the electron transportation [84]. Furthermore, there are possibilities to amplify the boundary scattering of phonons without increasing the electron scattering and thus the ZT of the thermoelectric devices can be improved. 

In the previous section, it is anticipated that conductive polymers can be potential as thermoelectric materials, given their low thermal conductivity and improved electrical transport through doping with different nanoparticles [85]. This potential can be further exploited by fabrication of these polymer thermoelectrics in the form of nanoscale [86], through the electrospinning method, chemical vapor deposition method, electrodeposition method, and inkjet printing method.

## 4. Fabrication Methods for Thermoelectric Polymer Devices

### 4.1. Electrospinning

**Figure 11:**
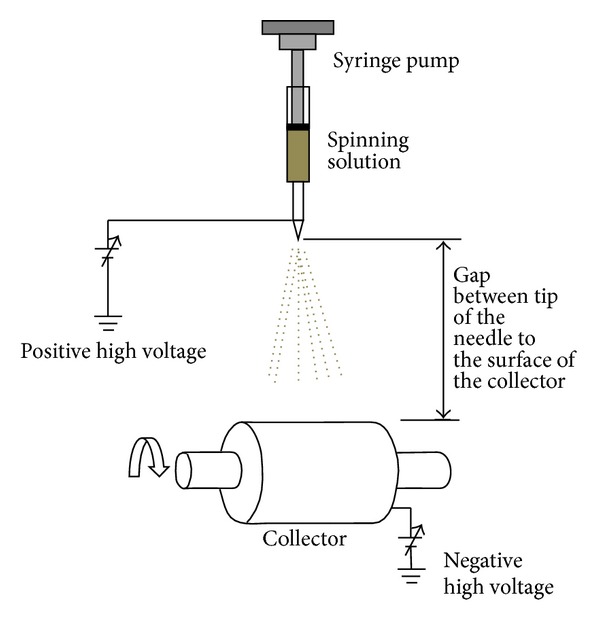
A basic electrospinning setup [88].

The electrospinning technique is a simple and elegant method to produce nanofibers. In 1934, Formhals patented a process to produce polymer filaments using electrostatic force. Later on, the process evolved and was named as electrospinning [79–81]. This method is able to produce continuous nanofibers from polymer solutions or melts in the presence of high electric fields, in the region of 10–30 kV. So far, hundreds of polymers have been successfully fabricated by electrospinning process [87]. Thermoelectric polymers like carbon nanotube (CNT)/polyaniline (PANI) composite, polyaniline/(polystyrene and polyethylene Oxide) blends, polyaniline, polypyrrole, and polycarbonate nanofibers have been successfully fabricated through electrospinning process [57]. The basic schematic setup for the electrospinning process is shown in Figure 11. 

**Figure 12:**
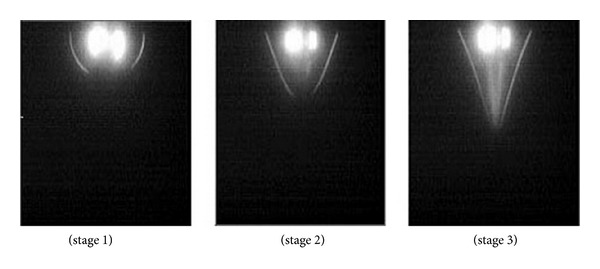
Formation of the Taylor cone [89].

In the electrospinning process a charged liquid polymer solution is introduced into an electric field. A high voltage (HV) direct current (DC) power supply is used to generate the potential differences in the range between 10 and 30 KV. A needle attached to a syringe is used to dispense the liquid polymer solution at a desired voltage between 10 and 30 kV. After that it is deposited on a collector which is grounded. The cathode of the HV power supply is attached to a wire and inserted into the syringe containing the polymer solution and the anode is attached to the ground. A rotating drum, usually wrapped with aluminum foil can be used as a collector. The tip to collector distance is maintained between the ranges of 10–30 cm. The inner diameter of a needle can be between 0.5–1.5 mm. The ejected polymer solution forms a continuous nanofiber when the high voltage overcomes the surface tension. Once the ejection starts, at the tip of the needle, the pendant droplet of the polymer solution forms a conical shape, typically referred to as Taylor cone. Whilst the fluid is charged, the surface charge and the surface tension operate in opposite relation. Therefore, the fluid changes shape and the formed structure is known as the Taylor cone [89]. The images of the formation of a Taylor cone are shown in Figure 12.

**Figure 13:**
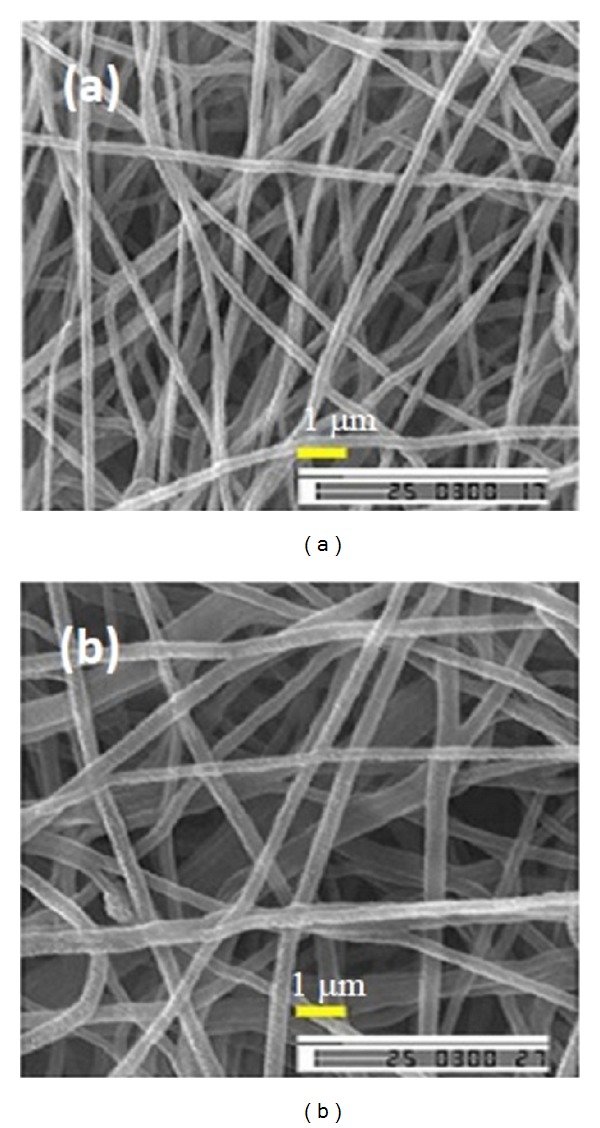
SEM micrographs of a polypyrrole electrospun nanofibers, formed from aqueous solutions of 1.5 wt% poly(ethylene oxide) as carrier, with (a) and without (b) 0.5 wt% Triton X-100 surfactant. The polypyrrole content of the nanofibers is 71.5 wt% [91].

The key parameters which affect the formation of nanofibers are (1) solution parameters such as viscosity, conductivity, surface tension, and vapor pressure; (2) process parameters such as shape of collector, needle diameter, solution flow rate, tip to collector distance, and applied voltage; (3) ambient parameters such as solution temperature, humidity, and air velocity in the electrospinning chamber. By varying these parameters the thickness and smoothness of the fibers can be controlled [90]. A typical SEM micrograph of an electrospun nanofiber (Polypyrrole) is shown in Figure 13.

### 4.2. Inkjet Printing

Recently the inkjet printing approach has been adopted for fabrication of electronics devices, where the colored ink cartridge is replaced with a functional electronic ink, for example, semiconducting ink and conductive ink. Today, inkjet printing is used in electronic industries and research for fabrication of printed circuit boards (PCB), light-emitting diodes (LED), thin film transistors (TFT), solar cells, thermoelectric devices, and many others [91–96]. 

**Figure 14:**
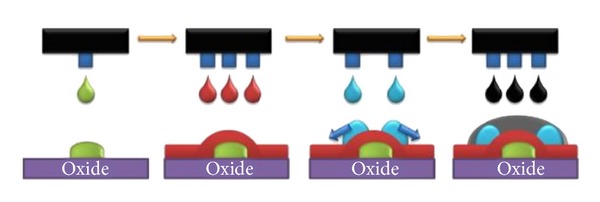
Step by step process for fabrication of device using inkjet printing [98].

With that being said, inkjet printing is an attractive method to be used in the fabrication of the whole or part of the structure of thermoelectric devices. Traditionally, thermoelectric devices structure is produced by conventional printing such as lithography and roll-to-roll printing [97]. Conventional methods require series of processes flow such as etching, cleaning, and dicing, which is time consuming and produces a lot of waste material [91]. With the ability to accurately deposit minute amounts of materials onto substrate, inkjet printing provides the alternative for fabrication of thermoelectric devices. A schematic of the operation of the inkjet printer is shown in Figure 14. Ink is deposited onto substrate to fabricate multiple layers structure. 

The field of printable electronics is becoming more significant. This technology requires the active electronic materials, such as metals, organometallics, nanoparticles, and biopolymers in solution form, and thus soluble organic or polymeric materials are attractive candidates for this processing method. The inkjet printing method is also tuned for room temperature processing, and only a small amount (in the region of picoliters for a cartridge) is required to produce a device, thus resulting in substantial cost reductions [91]. Ink is directly and accurately deposited onto various types of substrates, and multilayer and planar multicomponent systems are able to be fabricated with this technique [99]. High resolution, in the region of 20–50 micrometers is possible, and no masks are required to form the required device design [91, 96, 100]. As such, on-demand production is possible. 

**Figure 15:**
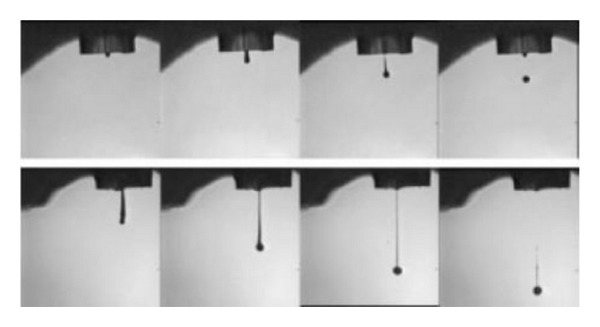
Stroboscopic images of droplets produced by inkjet printing [91].

Printing quality is strongly influenced by the ink properties. Printing resolution depends on the ink viscosity and surface tension [96]. The viscosity must be low enough to allow the channel to be refilled in about 100 *μ*s of the droplet dispensed time lapse (deposition and dispensing rate).  Figure 15 shows the droplet formation by inkjet printing. To hold the ink without dripping, the ink must possess high surface tension and low pressure. Dispensed droplet energy goes into viscous flow, surface tension, and kinetic energy of the drop [91, 100]. 

Inkjet printing inks including sol-gel, conducting polymers, ceramics, metals, nanoparticles, and biopolymers ink have been used widely for various inkjet-printed devices [91, 100]. The physical properties of ink play important role in inkjet printing technology. For example, a low ink viscosity of about 20 cP is favorable in order to have the appropriate amount and velocity of ink ejected from the nozzle. Surface tension of the ink will determine the spheroid shape of the ink after ejection, and is typically between 28 mN·m^−1^ and 350 mN·m^−1^. Molten metals typically have very high surface tension; meanwhile, structural polymers possess very low surface tension [91, 100]. Polymeric additives are used to improve dye bonding to the substrate and improve the ink viscosity. Humectants such as ethylene glycol are added at 10%–20% to prevent clogging and drying of the nozzle. The surface tension of the ink is advised to be at a minimum of 35 mN·m^−1^.

**Figure 16:**
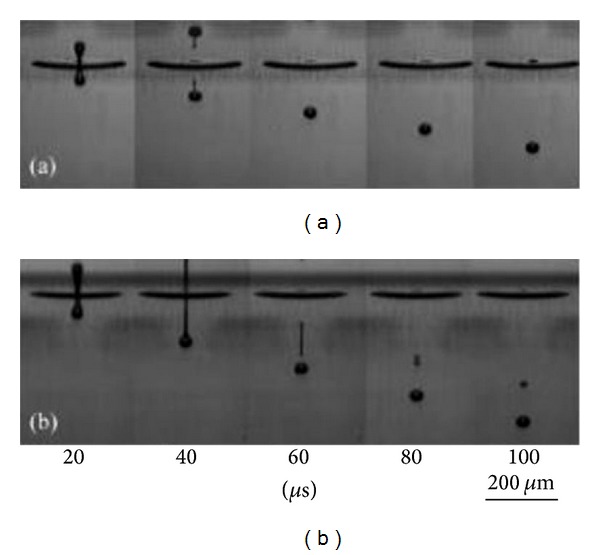
Comparison of (a) PEDOT and (b) F8 ink droplets [94].

Examples of conducting polymers commonly used in inkjet printing are polyaniline (PANI) and PEDOT : PSS [92].  Figure 16 shows the inkjet profile of poly(ethylenedioxythiophene) (PEDOT) and poly(9,9-dioctylfluorene) (F8) aqueous solution being deposited onto a prepatterned substrate.  Figure 15 illustrates the impact of the ink's physical properties on the droplet profile. The formation of F8 long tail is seen from the stroboscopic image. The difference in profile is caused by the difference in viscosity, surface tension, and the polymer molecular weight of the respective solutions. 

Printing quality is also controlled by substrates surfaces and ink interaction [91, 101]. Chemical modifications of substrates are a common practice in improving printing quality. The hydrophilic character of substrates surfaces is controlled to prevent excessive absorption of liquids and inks. Surface porosity and roughness influence ink spreading onto the substrate. Synthetic polymer is produced to form a thin film on the substrates surfaces which promote better inkjet printing quality. Substrate treatments are found to be important in keeping excellent printing quality. It must be performed such that hydrophilic character of substrate is kept to allow surface wetting whenever necessary [101].

As is the case with polymeric thermoelectric materials in general, the inkjet printing process is best suited for low temperature thermoelectric materials, for applications in ambient temperature such as hybrid solar cells, and body heat electricity generation. Even higher resolutions may be attained using electrohydrodynamic jet printing, which allows for high resolution, precision, and speed printing [102]. Jet printing generates small scale droplets targeted on nano- and microscales researche. The use of ultrafine inkjet printer allows a minimum size of dots of less than one micron, thus allowing fabrication of materials and devices using inkjet printing, which enter the realm of nanotechnology [103].

Thus, given the attractiveness of the inkjet printing method, that is, solution processability, low amounts of ink, direct patterning of device structure onto substrate, the possibility of flexible substrates, and on-demand fabrication, there is much potential for fabrication of thermoelectric devices using inkjet printing.

### 4.3. Chemical Vapor Deposition

In 1890, Mond, Langer, and Quincke developed the Chemical Vapor Deposition (CVD) technique for large scale applications as a carbonyl process to refine nickel. Numerous earliest applications were implicated in refining or purification of metals and a limited number of nonmetals by carbonyl or halide processes. In recent times, CVD, research, and development efforts have been more concentrated towards the thin-film deposition. It is actually widely used in materials-processing technology. Mostly, it is involved in solid thin-film coatings to surfaces. However, this technique is recurrently used in producing carbon nanotubes (CNTs) high-purity bulk materials and powders, as well as fabricating composite materials via infiltration techniques. So far, the majority of the elements in the periodic table have been deposited by CVD techniques, with some being in the form of the pure elements, but mostly in combinations to form compounds [104].

**Figure 17:**
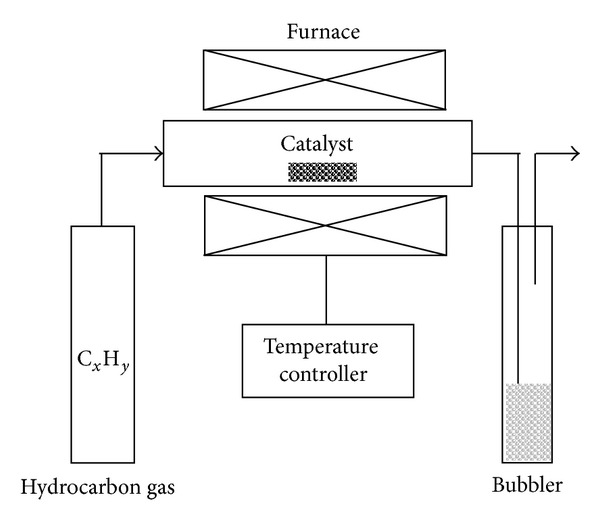
Schematic diagram of a simple CVD setup [106].

In CVD process, precursor gases are brought into a reaction chamber in an activated (light, plasma, and heat) environment and directed towards a heated substrate. Thus, a controlled chemical reaction is induced. The chemical reactions result in the deposition of a solid thin film material onto the substrate surface. It is a very useful processing method for the deposition of polycrystalline, amorphous, and single-crystalline thin films and coatings for a wide range of applications [105].  Figure 17 shows the basic schematic diagram of a CVD setup.

**Figure 18:**
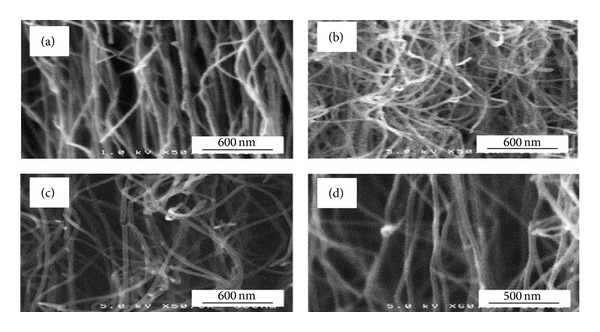
High magnification SEM pictures of vertically aligned CNTs on samples ((a)–(d)) indicating the different degrees of tube alignment [108].

A high-temperature tube furnace with a quartz tube can be used to fabricate nanofibers at temperatures ranging from 300°C to 1200°C. The reaction duration can be varied in the range of 15 min to 8 h according to the desired length of nanowires and thickness of the thin films. The synthesis can be regulated through several parameters such as hydrocarbon's concentration, catalyst, temperature, pressure, gas-flow rate, deposition time, and reactor's geometry. Recently, CVD has attracted a lot of attention in producing CNTs through thermal CVD or catalytic CVD (to distinguish it from many other kinds of CVD used for various purposes) [106]. Carbon nanotubes show high electron mobility and high electrical and thermal conductivity and are able to sustain a huge amount of current before structural failure. These properties along with their high Seebeck coefficients make them superlative candidates for thermoelectric applications [107]. SEM images of vertically aligned CNTs fabricated through thermal CVD are given in Figure 18.

### 4.4. Electrochemical Deposition

Electrochemical deposition or in short electrodeposition has been used in producing thin films and has been intensively used for the last 35 years [109]. It is a nonvacuum, easily scalable, cost-effective, and room temperature technique which made it a more convenient option. Moreover, differently shaped and sized substrates can be used in this method and in contrast to the other gas phase techniques, toxic gaseous precursors are not needed [110]. So far, several thermoelectric thin films have been fabricated through this method. This technique has been used in fabricating both organic and inorganic thin films [111].

This technique is used as an electrochemical liquid phase thin film preparation method. Usually, the design of an electrochemical cell depends on the specific needs of the experiment. In general, 25–50 mL cell can be used for the laboratory experiments for the sake of handiness. In this process, the reactions are either reduction or oxidation, completed by using an external current source. To carry out the deposition process, an electrochemical cell consists of a reaction vessel and two or three electrodes. In the three-electrode cell, a reference electrode is used to control or measure the potential of the working electrode. However, the current passes between the working electrode and a separate auxiliary or counter electrode. Depositions are controlled by regulating either current or potential. All the compartments of the cell can be separated by using glass frit to reduce the interference of electrochemical reactions. This setup is used when the cell resistivity is relatively higher. The working electrodes work as cathode. Gold, platinum, carbons, mercury, and some semiconductors can be used as working electrodes. Most commonly, reference electrode is “Saturated Calomel Electrode (SCE)” or the Ag/AgCl electrode. Platinum wires or mesh can be used as counter electrodes or anodes. The reactions can occur in room temperature [112]. 

**Figure 19:**
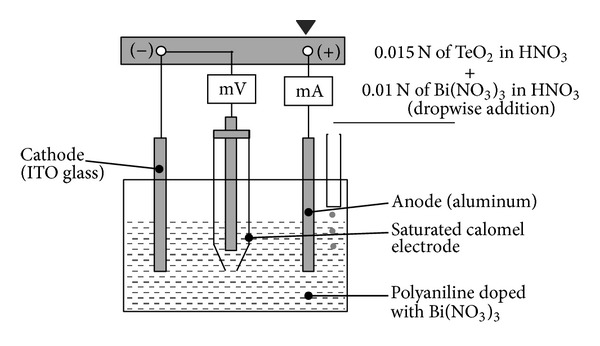
The schematic representation of electrochemical deposition system for PANI/Bi_2_Te_3_ [113].

**Figure 20:**
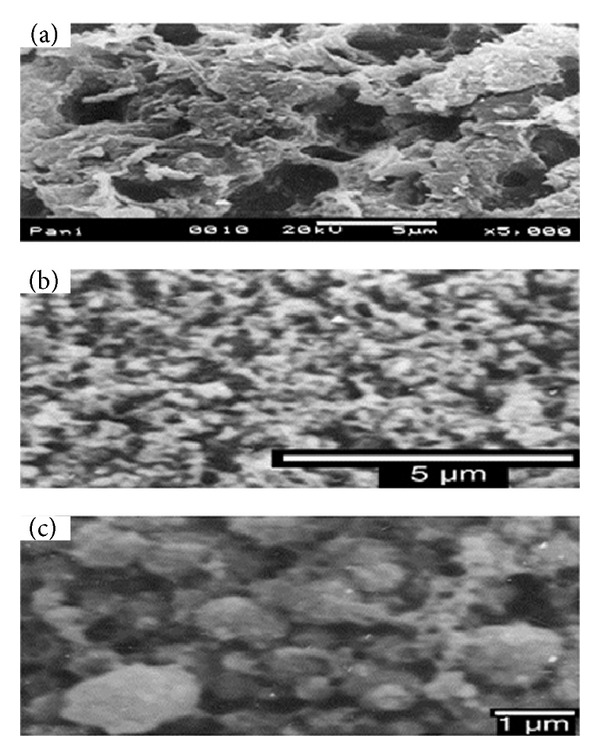
SEM images of (a) pure polyaniline, (b) PANI/Bi_2_Te_3_, and (c) PANI/Bi_2_Te_3_ [113].

Recently, thermoelectric material composed of conductive polymer polyaniline (PANI) and Bi_2_Te_3_ nanocomposites was prepared using a simultaneous electrochemical reaction and deposition method. The three-electrode system was used in the fabrication process (Figure 19). After deposition, smaller molecules of Bi_2_Te_3_ were found to be dispersed in the macromolecules of PANI [113]. SEM images of samples are prepared by the electrochemical deposition system (Figure 20).

## 5. Future and Challenges of Thermoelectric Polymers

The developments of polymer thermoelectric materials have seen dramatic increase over the last half decade. Despite that, there is reasonable development to be carried out before real commercialization of polymer-based thermoelectric devices can be realized. Low ZT renders the material suitable for small device applications as these materials consume less energy. Normally, the efficiency of a real device will be much lower than that fabricated in the lab. This can be attributed to the fact that the synthesis is usually not reproducible and sometimes only a small amount could be synthesized and there will always be a problem of large variations in performance between different batches of materials. Second, during manufacturing process, a lot of starting materials are wasted as the most effective fabrication method is still being developed. This contributes to the high cost of manufacturing and thus not favorable to the manufacturer. In order to address this problem, researchers have started to use the inkjet printing technique where only a small amount of thermoelectric sample, in the range of picolitres, is needed to fabricate a device. The resolution of the inkjet printer is suited to those within the range of a few micrometers if only, the inkjet printer is able to print with a resolution as small as nanoscale [114]. 

There have been a few drawbacks in polymers to be potential TE materials thus far. Lower Seebeck coefficients and lower electrical conductivity are yet to address to employ the polymer TE material as efficient TE device. Low Seebeck coefficient is improved by the introduction of nanostructures. In addition, the effect of humidity on electrical conductivity is also a constraint to increase the ZT of polymer TE materials as additional treatment in fabrication and encapsulation of the TE device is required in order to mitigate the humidity effects. Summarily, conductive polymers such as PEDOT : PSS and PEDOT : TOS [109] mixtures have been responsible for the fast trajectory in TE performance in the class of organic electronics, where the ZT has increased from ZT ~ 10^−6^ to ZT = 0.45 within the space of 15 years and is a promising indicator that the performance of polymer TE may be close to bridging the gap in performance compared to inorganic TE materials and it will be viable for commercial equations [115].

### 5.1. Next Generation Dye-Sensitized Solar Cells (DSSCs)

For the past few decades, research on dye-sensitized solar cells (DSSCs) has attracted the attention of many academics due to the simple fabrication process, low cost, and potentially high efficiency of converting sunlight energy to electric energy [116–119]. The DSSC architecture was first reported by the Grätzel group who successfully developed a DSSC with power conversion efficiency (PCE) of 11% using a liquid electrolyte [120]. The DSSC consists of four major components: (i) a working electrode, (ii) a layer of titanium dioxide (TiO_2_) coated with dye, (iii) electrolyte, and (iv) a counter electrode. Photons from sunlight are absorbed by the dye. Once the dye is excited, electrons escape from the dye and are accumulated in the nanocrystal TiO_2_ layer. Due to the large amount of energy lost in the form of heat, the power produced is usually lower than the energy absorbed by the system. The electrons are then diffused into the electrode and eventually return back again into the dye through electrolyte. These processes not only generate electricity but also create a temperature difference where the working electrode is the cool side and the counter electrode is the hot side. In addition, photons with lower energy may be absorbed by glass substrate, electrolyte, and electrodes and convert to heat. Thus, the working temperature of DSSC may be as high as 60°C or higher.

**Figure 21:**
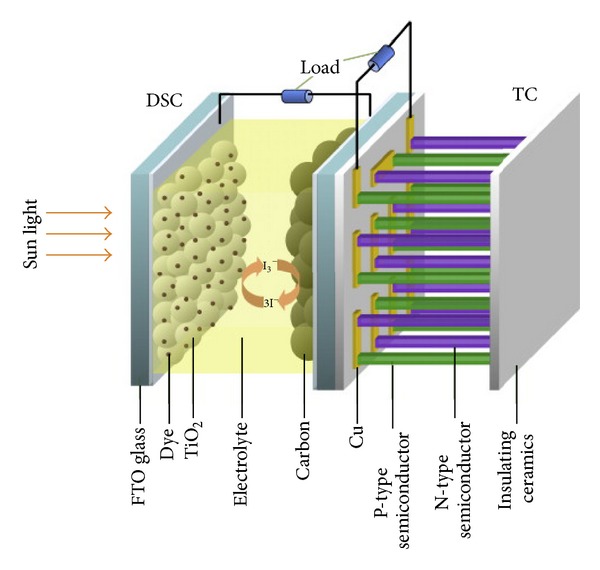
Schematic structure of TE generator-DSSC hybrid system [124].

There have been many efforts have been applied to TE materials to harvest electricity from solar energy [121, 122]. For the past few years, there have been efforts to combine thermoelectricity with DSSC and utilizing the waste heat produced by DSSC to generate electricity [123]. This device is expected to have an improved the overall efficiency. In 2010, Guo et al. [124] fabricated a TE cell-DSSC hybrid system by placing the back of the DSCC to the hot side of the TE cell. They achieved an improvement of 10% of the overall PCE by using the hybrid system compared with a single DSSC. In the hybrid system, DSSC absorbs photon and generates electricity and heat, whilst the TE cell utilizes this waste heat to convert to electric power. One of the interesting parts of this device is that the two subcells can either work at the same time or one at a time as shown in Figure 21.

Wang et al. [125] found that by adding a solar selective absorber (SSA) to the TE cell-DSSC hybrid system, the conversion efficiency can be further enhanced. SSA can be a material or coating that can maximize solar absorption and minimize thermal emission. Examples of SSA material are PbS, NiCrO_*x*_, NiS-ZnS, and Cr-Cr_2_O_3_. The solar energy transmitted through the DSSC is absorbed by the SSA which will convert the energy into heat. This heat is then converted into electricity using the Seebeck effect. The maximum PCE achieved was larger than 13% which shows a promising configuration for realizing very high conversion efficiencies.

## 6. Conclusions 

The developments of polymer thermoelectric materials have seen dramatic increase over the last half decade. The applications of thermoelectric polymers at low temperatures, especially conductive polymers, have shown various advantages compared to inorganic materials such as easy and low cost of fabrication, light weight, flexibility, and low thermal conductivity. However, thermoelectric polymers have shown some drawbacks such as low electrical conductivity and Seebeck coefficient. Nanostructuring, polymer composites, nanotubes, and addition of semiconducting stabilizers have been shown as important approaches to improve the performance of thermoelectric polymers. By taking advantage of different fabrication techniques, the morphology and structure of the thermoelectric materials and devices can be fine-tuned to suit the intended application. Not only that, but also the control of the morphology will allow for an improvement in the performance of the thermoelectric materials. The use of thermoelectricity with on dye-sensitized solar cells and utilizing the waste heat produced by on dye-sensitized solar cells will be a promising technology to generate electricity from solar energy and low temperature heat sources. 

## Figures and Tables

**Table 1:** Thermoelectric property of various polymers.

Polymer	Conductivity *σ* S/cm	Seebeck coeffient S *μ*V/K	Thermal conductivity *κ* W/mK
Polyacetylene [33–36]	~1.53 × 10^−3^–2.85 × 10^4^	~−0.5–1077	—
poly(*p*-phenylene vinylene) [37, 38]	10^−5^	7	7.2 × 10^−11^
Polyaniline [37–39]	7000	7	5.1 × 10^−2^
poly(2,7-carbazolenevinylne) [37, 38]	5 × 10^−3^	230	8.0 × 10^−5^
poly(2,5-dimethoxyphenylenevinylene) [40]	46.3	39.1	—
